# Performance Enhancement of the Passive Heat Exchanger in the MNTZV-159 Metal Hydride Storage System Using Triply Periodic Minimal Surface (TPMS) Structures

**DOI:** 10.3390/ma19163539

**Published:** 2026-08-20

**Authors:** Šimon Hudák, Marián Lázár, Gabriela Ižaríková, Tomáš Brestovič, Natália Jasminská, Peter Čurma, Romana Dobáková, Peter Milenovský

**Affiliations:** 1 Department of Power Engineering, Faculty of Mechanical Engineering, Technical University of Košice, 04200 Košice, Slovakia; simon.hudak@student.tuke.sk (Š.H.); tomas.brestovic@tuke.sk (T.B.); natalia.jasminska@tuke.sk (N.J.); peter.curma@tuke.sk (P.Č.); romana.dobakova@tuke.sk (R.D.); peter.milenovsky@tuke.sk (P.M.); 2 Department of Applied Mathematics and Informatics, Faculty of Mechanical Engineering, Technical University of Košice, 04200 Košice, Slovakia; gabriela.izarikova@tuke.sk

**Keywords:** metal hydride, heat exchanger, TPMS structures, storage system, hydrogen

## Abstract

**Highlights:**

- A new design of a Diamond TPMS heat exchanger was created for a low-pressure metal hydride storage tank.
- In the improved TPMS heat exchanger, the average hydride temperature decreased by 21.5 °C.
- In the TPMS heat exchanger, passive heat removal was enhanced while the hydrogen storage capacity was preserved.

**Abstract:**

Low thermal conductivity of metal hydride beds significantly limits the hydrogen absorption kinetics and performance of metal hydride storage systems. This study presents a new design of a passive internal heat exchanger for a certified MNTZV-159 low-pressure hydrogen storage tank using the Triply Periodic Minimal Surface (TPMS) structures. The parametric improvement in the design of a cylindrical Diamond TPMS-based geometry was performed by applying various cell dimensions, arc counts, and wall thicknesses while maintaining the original volume of the heat exchanger. The analysed configuration was subsequently evaluated through three-dimensional numerical heat-transfer simulations conducted in ANSYS CFX. Compared with the original finned heat exchanger, the TPMS-based design reduced the average metal hydride temperature from 107.7 °C to 86.2 °C and the maximum temperature from 130.9 °C to 114.0 °C. The improved temperature uniformity enhanced the heat removal from the hydride bed and created more favourable conditions for hydrogen absorption. The results demonstrated that TPMS structures constitute a promising solution for improving passive thermal management in metal hydride hydrogen storage systems while maintaining the storage capacity of the vessel.

## 1. Introduction

Hydrogen may be regarded as one of the key elements to be used in the future low-carbon energy industry, with potentially strategic importance with regard to the decarbonisation of the energy industry and other industries. At present, hydrogen technologies, a promising tool for the transformation of the energy sector towards a sustainable economy, are still in the stage of development and gradual implementation. More extensive deployment of these technologies requires strategic investments and further technological, economic, and infrastructural development [1]. The most fundamental barriers to the competitiveness of hydrogen technologies include high energy intensity of hydrogen production, high investment costs associated with hydrogen production, storage, and distribution, insufficiently developed infrastructure, as well as technical challenges related to hydrogen storage safety and effectiveness [2,3,4,5].

With regard to safety and the ability to maintain operational pressure at low levels, the storage systems based on the formation of metal hydrides are especially promising. Hydrogen is stored reversibly in a metal or intermetallic matrix through a chemical reaction described as follows (1) [6]:

(1) $$M+\frac{x}{2}H_{2}↔MH_{x}+\Delta H$$

where M is a metal or an alloy capable of binding hydrogen; H_2_ is molecular hydrogen in the gaseous phase; MH_x_ is the formed metal hydride; x is the number of hydrogen atoms bound to a single atom of metal; and Δ*H* is the reaction heat (J·mol^−1^).

Despite the fact that many hydrides have favourable thermodynamic properties, their practical usability, in terms of their real absorption kinetics in storage tanks, is significantly limited by low effective thermal conductivity of the metal hydride bed. This notably increases the refuelling time and reduces their competitiveness compared to that of high-pressure systems. Thermal conductivity of powder metal hydride materials is often defined with a value of ≤1 W·m^−1^·K^−1^; as a result, significant temperature gradients are formed in the volume of MH materials [7]. Accordingly, hydrogen absorption in the hydride volume is uneven. During hydrogen absorption (an exothermic process), the temperature of the metal hydride alloy and of the storage system itself rapidly increases. The temperature increase causes an increase in equilibrium pressure, as described by the Van’t Hoff equation, while the amount of stored hydrogen decreases or the absorption process stops [6,8,9,10].

(2) $$\ln p=\frac{\Delta H}{R\cdot T}-\frac{\Delta S}{R}$$

where *p* is the equilibrium pressure of hydrogen above metal hydride (Pa); Δ*H* is the enthalpy of hydrogen production/decomposition of Hydralloy C5 hydride (−22,540 J·mol^−1^); Δ*S* is the reaction entropy (−97.23 J·mol^−1^·K^−1^); R is the gas constant (R = 8.314 J·mol^−1^·K^−1^); and *T* is the thermodynamic temperature (K).

The inversion process is detectable during the process of hydrogen desorption, in which the storage system (a metal hydride material) consumes heat, which must be supplied to the system. Insufficient heat supply causes a decrease in temperature and equilibrium pressure. As a result, the flow rate of released hydrogen decreases. Due to the thermodynamic reasons applicable to the absorption–desorption cycle, the speed of fuel (H_2_) storage and release is limited by the efficiency of the thermal management system in the metal hydride storage system, i.e., by the system’s ability to remove and supply reaction heat [11].

Geometric modification of the metal hydride storage tank, or the storage system, is the first step in the process of enhancing heat conduction in the metal hydride (MH) bed. Depending on the type of thermal management system (active or passive), the heat-transfer surfaces in the MH bed, which remove and supply thermal energy in order to maintain the highest possible efficiency of the system, are extended. The most frequently applied method for improving heat transfer in the MH bed is to integrate heat exchangers into the metal hydride volume. The fins of the internal heat exchanger reduce local overheating of the MH bed, while the distance to which heat is conducted from the core of the storage tank towards its shell is reduced. Despite the high efficiency of internal heat exchangers, they have certain disadvantages, including an increase in the weight of the storage system, a lower volume in the storage tank available for metal hydride, and a more complicated process of filling and potentially replacing the MH bed. Moreover, an increase in the number of weld joints on MH storage tanks increases the risk of potential hydrogen escape points and causes hydrogen embrittlement [8,12].

More complex heat-transfer surfaces can be produced using additive manufacturing technologies, such as SLM and DMLS. They facilitate the formation of very complex geometries made of aluminium, copper, or steel, which could not be produced using conventional manufacturing technologies. For manufacturing purposes, under forced-convection cooling, it is possible to optimise porosity, wall thickness, and cell size, or locally adjust the geometry based on the required thermal conditions [13].

TPMS architectures have demonstrated considerable potential in compact heat exchangers. The closest published studies are dominated by purpose-built gyroid reactors. Lesmana and Aziz reported that forced-convection cooling reached 90% hydrogen absorption in 2000 s at a heat-transfer coefficient of 500 W·m^−2^·K^−1^, whereas natural convection required almost twice as long [14]. In a subsequent experimental study, a gyroid reactor filled with LaNi5 reached its full hydrogen capacity within 1020 s at a charging pressure of 0.5 MPa under room-temperature cooling [15]. Jiang et al. later achieved a hydrogen absorption volume fraction of 0.926 for a TPMS-equipped reactor in a coupled numerical model, exceeding the corresponding finned configuration [16]. These studies demonstrate the thermal potential of TPMS, but they do not address a volume-neutral replacement of the internal heat exchanger in an already certified pressure vessel.

An innovative approach to designing compact heat exchangers involves the use of Triply Periodic Minimal Surfaces (TPMSs). Compared to conventional finned exchangers, the TPMS geometries form continuous curved surfaces without sharp edges, and this reduces the formation of thermal boundary layers [17]. In a paper by Khaoula Amara et al., the use of TPMS structures for coolers and heat exchangers is analysed [13]. The authors showed that the Gyroid, Diamond, and Schwarz-P geometries facilitate achieving more intensive heat transfer, better thermal homogeneity, and as much as a 25–30% higher PEC (Performance Evaluation Coefficient) value compared to those achieved with conventional finned heat exchangers. Mohamed G. Gado and his team analysed the design, production, and thermal applications of TPMS structures [17]. They emphasised their high surface area-to-volume ratio, favourable mechanical properties, and the potential for applications in heat exchangers, for heat accumulation, cooling of batteries, and in hydrogen systems. In their paper, Ruochao Zhao et al. analysed the integration of Gyroid TPMS structures in a hydrogen storage reactor [18]. Their results showed that the implementation of TPMS structures improved the removal of reaction heat and accelerated the process of hydrogen absorption by 23.4% compared to a conventional solution. TPMS heat exchangers are a promising alternative to conventional finned exchangers.

Another method of increasing the efficiency of heat removal is to increase the effective thermal conductivity of the metal hydride bed through mixing it with conductive materials (expanded graphite, carbon nanomaterials, metal powders and fibres), or through using aluminium foam, copper foam, or a graphite matrix for heat transport from the core of the metal hydride bed towards the storage tank walls [8]. Laurencelle et al. proved an increase in effective thermal conductivity from approximately 0.15 W·m^−1^·K^−1^ to approximately 10 W·m^−1^·K^−1^, which was achieved after integrating aluminium foam into the LaNi_5_ bed [19]. Mellouli et al. observed that the use of metal foam facilitated an approximately 60% reduction in the time required to achieve 90% saturation of the metal hydride bed [20].

Over the last few years, heat pipes (HPs) have also been subjected to intensive examination. HPs are passive devices able to transport large heat fluxes at a minimum temperature difference. Their advantage is very high effective thermal conductivity and the absence of moving parts [21]. Multiple studies showed that a combination of heat pipes and fins or metal foam led to a significant increase in storage tank performance [21].

Another interesting solution aimed at increasing the efficiency of a thermal management system for metal hydride (MH) storage tanks is the use of phase change materials (PCMs) [21,22]. These materials absorb and release large quantities of latent heat at almost a constant temperature of the MH bed. A team of authors, Hasnain et al., stated that optimally designed PCM systems may reduce the absorption time by dozens of percent [23]. Powder metal hydride alloys and phase change materials still exhibit low thermal conductivity, which may be increased by using expanded graphite, metal foams, finned structures, or heat pipes [8,21].

In a subsequent study, the authors demonstrated that the effective thermal conductivity of spinodal metamaterials can be deliberately and directionally tuned by modifying the parameters of the Cahn–Hilliard equation. Furthermore, anisotropic structures exhibited a strong, approximately linear relationship between thermal conductivity and Young’s modulus. The numerical models and the derived thermomechanical cross-property relations (CPRs) were successfully validated experimentally. FE predictions using UTG boundary conditions deviated from the experimental measurements by less than 5%, while CPR-based predictions showed a maximum deviation of approximately 7% [24].

Based on the available scientific results, there is no universal optimal solution to designing a thermal management system for MH storage tanks. The suitability of any designed methodology for supplying and removing thermal energy from the absorption–desorption cycle is determined by the type of application in which the storage tank is to be installed (laboratory conditions, industry sector), boundary conditions defined by the utilisation method, and the type of MH bed. Where an MH storage tank is installed in a real-life application, its design and manufacture will be subject to limitations stipulated by related legislation, decrees, and technical norms.

The available results therefore do not identify a universally optimal thermal management architecture for metal hydride storage. Optimal solutions vary, depending on the hydride, operating conditions, allowable auxiliary power, vessel geometry, and the fraction of internal volume that may be displaced by the heat-transfer structure. In an existing certified vessel, the design space is narrower because the pressure boundary, shell heat exchanger, connection layout, and usable hydride volume cannot be changed freely [11,25]. Comparison with the closest TPMS and MNTZV-159 reactor studies is shown in Table 1.

The present scientific paper deals with the redesign of an aluminium heat exchanger for a certified MNTZV-159 storage tank while respecting the limitations. The process of generating the geometry was limited by the existing cylindrical shell and the volume of the original aluminium heat exchanger. Structural improvements in the design consisted of the creation of TPMS with external edging aimed at enhancing the heat transport between the heat exchanger and the tank shell, which had a larger heat-transfer surface area at the same volume of the heat exchanger, and in the reduction in the average distance between the MH grains and the heat-transfer surface of the exchanger. The resulting scientific benefit lies in a quantified enhancement of the heat output that may be achieved while respecting the geometric and volume-related limitations relevant to the certification.

## 2. Materials and Methods

**Figure 1:**
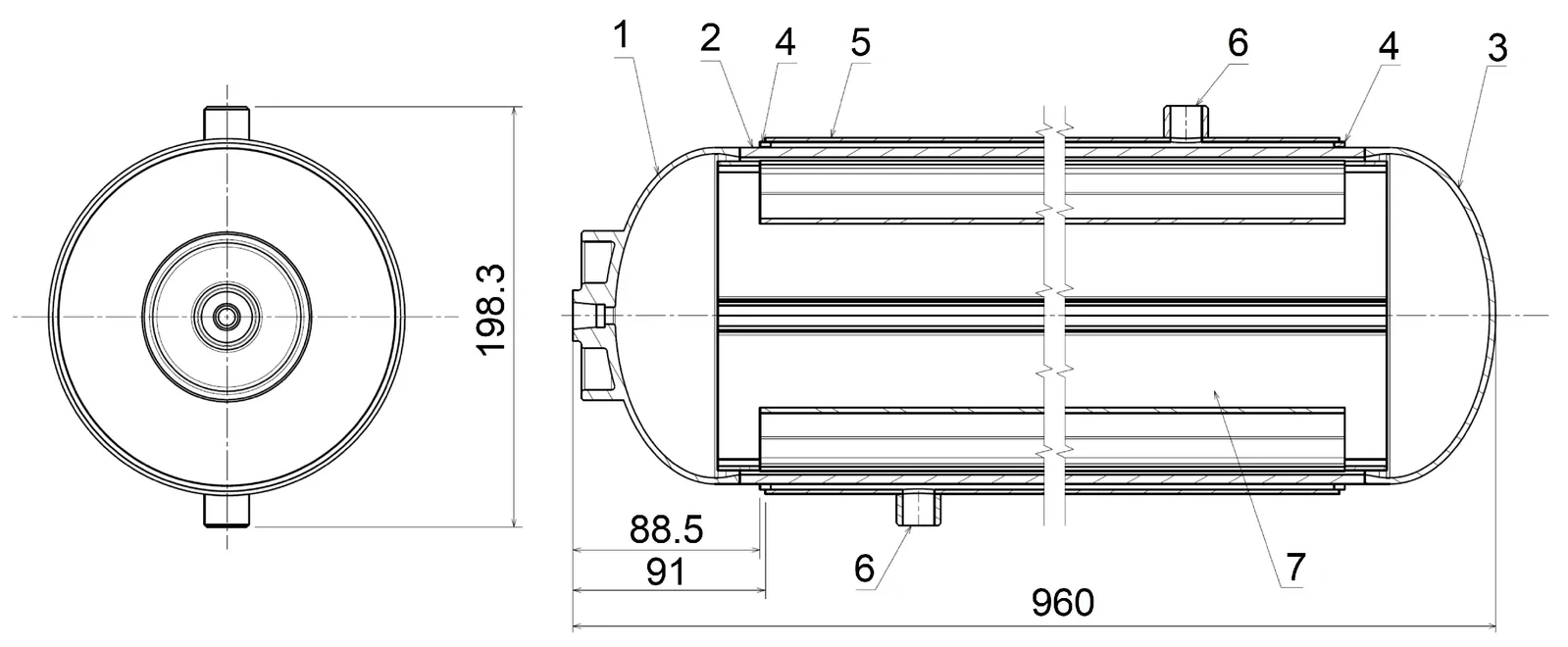
Drawing of the MNTZV-159 low-pressure storage tank: 1—elliptic bottom (159 mm × 79 mm; segment diameter × segment length); 2—cylindrical part of the primary storage tank (159 mm × 819 mm); 3—elliptic bottom (159 mm × 62 mm); 4—annular ring (164.3 mm × 5 mm); 5—cylindrical part of the jacket (168.3 mm × 795 mm); 6—socket; and 7—heat exchanger.

The focus of the investigation presented in this paper was the enhancement of heat transfer in the internal passive heat exchanger installed in a certified MNTZV-159 low-pressure metal hydride hydrogen storage tank, which was developed at the Faculty of Mechanical Engineering of the Technical University of Košice, Slovakia. The MNTZV-159 storage tank is a double-shell commercial pressure device intended for low-pressure hydrogen storage through reversible absorption of gaseous hydrogen into an alloy that is able to form metal hydrides. Its structure consists of an internal pressure vessel, filled with a metal hydride material (Hydralloy C5, AMG Titanium GfE, Nürnberg, Germany), containing an integrated system for heat-transfer intensification, an external shell heat exchanger, and necessary process fittings, including filtering and closing components. The device was designed and manufactured in compliance with the requirements defined in STN EN 13322-2 [27] for a maximum operational pressure of 30 bars, and its storage capacity is 0.65 kg of hydrogen (Figure 1) [21].

The modular structure of the storage system facilitates flexible scaling of its total storage capacity based on the energy requirements of a particular application, for both stationary and mobile energy systems. This approach also creates preconditions for the effective integration of the metal hydride storage technology into decentralised hydrogen energy systems.

The key component of the analysed storage tank was an internal aluminium heat exchanger, which was used as an extended heat-transfer surface, integrated directly into the volume of the metal hydride bed. It was replaced with a passive heat exchanger with TPMSs. Due to limited effective thermal conductivity of the powder metal hydride bed, local areas with elevated temperatures were formed, mostly in the zones with a relatively long distance between the primary and secondary fins in the radial direction. This process typically increases thermal resistance and subsequently leads to the formation of thermal inhomogeneities in the active volume of the storage tank. In order to improve the heat distribution and reduce the temperature gradients in the central part of the metal hydride bed, transverse fins were installed in the structure of the internal passive heat exchanger.

**Figure 2:**
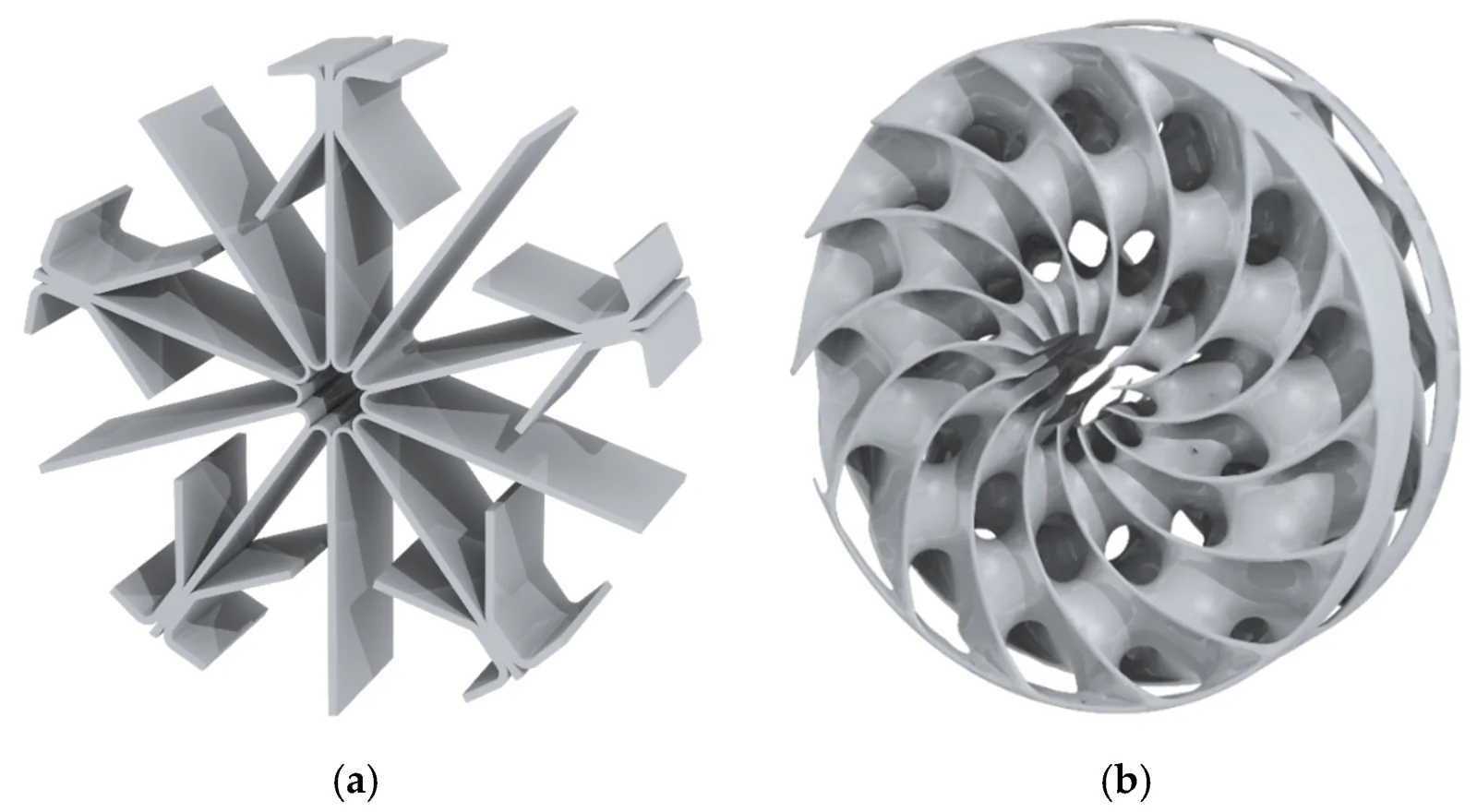
A 40 mm long geometry section of the original internal heat exchanger (**a**) and the new design (**b**).

The internal passive heat exchanger consisted of ten primary fins, while the secondary fins facilitated the enlargement of the total heat-transfer surface area. The secondary fins were installed in an alternate arrangement and attached to every other primary fin (Figure 2). The geometry contained a transverse fin with a length of 30 mm (part of the secondary fin), which formed a 35° angle with the primary fin. At the end of each secondary fin, a curved fin was formed in order to increase the intensity of heat transfer between the primary fin and the internal surface of the storage tank through the enlargement of the effective heat-transfer surface. In order to support the radial heat conduction, the cross-sectional surface area of the primary fin behind the first transverse fin was enlarged. The thickness of the primary and secondary fins (*δ*_1_) was 2 mm, while the thickness of the reinforced part of the primary fin behind the first transverse fin (*δ*_2_) amounted to 6 mm. The curved and secondary fins were not firmly attached to the cylindrical shell of the storage tank in order to maintain the structural integrity of the pressure vessel without having a negative effect on its strength and operational characteristics.

The replacement of the existing design was carried out with the use of the n-Topology software (version 5.40.2). The process of designing the new heat exchanger was conditioned by the following:

- The volume of the new internal heat exchanger should approach the reference volume of the original heat exchanger (93,126 mm^3^) in order to maintain the same metal hydride volume—the certification parameter of MNTZV-159;
- The distance between the internal wall of the storage tank and the external peripheral wall of the internal heat exchanger should be 1 mm.

The Triply Periodic Minimal Surface (TPMS) is known to have a large specific surface area and the potential to be used in thermal engineering applications. This study therefore examined and compared changes in the temperature in the MH alloy and the thermal field homogeneity following the integration of TPMS structures.

TPMSs, i.e., Triply Periodic Minimal Surfaces, are surfaces that occur periodically in three directions perpendicular to each other. At each point, they exhibit zero mean curvature, locally minimise their surface area, and divide the space into two interconnected channel systems which, however, do not cross each other. The best-known types of TPMSs include the Schwarz P (Primitive), Schwarz D (Diamond), Gyroid, Neovius, and others. The Diamond TPMS (Schwarz D) ranks among the best-known types of Triply Periodic Minimal Surfaces. The diamond structure creates a spatial mesh reminiscent of a diamond crystalline lattice. Thanks to its geometrical properties, it has no sharp edges or singularities, forms smooth connected passages, and exhibits a zero mean curvature and high spatial connectivity.

In the parametrisation of the internal heat exchanger, the correlations between the design parameters of the TPMS structure and the resulting values of the surface area and volume were taken into account. One of the common implicit equations for the Schwarz D surface area is as follows:

(3) $$\begin{array}{c} \sin\left(x\right)\sin\left(y\right)\sin\left(z\right)+\sin\left(x\right)\cos\left(y\right)\cos\left(z\right)+\cos\left(x\right)\sin\left(y\right)\cos\left(z\right)\\ +\cos\left(x\right)\cos\left(y\right)\sin\left(z\right)=0 \end{array}$$

Alternatively, it is often written as follows:

(4) $$\begin{array}{c} \sin\left(x\right)\sin\left(y\right)\sin\left(z\right)+\sin\left(x\right)\cos\left(y\right)\cos\left(z\right)+\cos\left(x\right)\sin\left(y\right)\cos\left(z\right)\\ +\cos\left(x\right)\cos\left(y\right)\sin\left(z\right)=t \end{array}$$

wherein parameter *t* determines a relative density of the structure.

The Diamond TPMS-based structure is typically defined in the Cartesian coordinate system (*X*, *Y*, *Z*). In applications onto objects with cylindrical symmetry, this structure may be subsequently mapped or transformed into a cylindrical coordinate system. The new passive heat exchanger was designed using a model created in the nTop environment with the use of cylindrical TPMS structures. In this case, the periodic diamond structure was generated in the cylindrical coordinate system (*r*, *θ*, *z*) with the aim of adjusting it to the cylindrical geometry of the model, while *r* is the distance from the cylinder axis, *θ* is the angle around the axis, and *z* is the height. In the definition of the TPMS-based structure in this system, the direction around the cylinder perimeter (*θ*) corresponded to parameter U, whereas the direction along the cylinder axis (*z*) corresponded to parameter V. The TPMS-based structure closures periodically occurred in the circumferential direction.

A general equation for a Diamond TPMS-based structure in the coordinates used in the Cylindrical Cell MAP (*U*, *V*, *W*) is as follows:

(5) $$\begin{array}{c} sin\left(\frac{2\pi U}{L_{U}}\right)sin\left(\frac{2\pi V}{L_{V}}\right)sin\left(\frac{2\pi W}{L_{W}}\right)+sin\left(\frac{2\pi U}{L_{U}}\right)cos\left(\frac{2\pi V}{L_{V}}\right)cos\left(\frac{2\pi W}{L_{W}}\right)\\ +cos\left(\frac{2\pi U}{L_{U}}\right)sin\left(\frac{2\pi V}{L_{V}}\right)cos\left(\frac{2\pi W}{L_{W}}\right)+cos\left(\frac{2\pi U}{L_{U}}\right)cos\left(\frac{2\pi V}{L_{V}}\right)sin\left(\frac{2\pi W}{L_{W}}\right)=C \end{array}$$

wherein *U* is the local coordinate in the direction of the Cell Radius; *V* is the local coordinate in the direction of the Cell Height; and *W* is the local coordinate around the perimeter (*L*_W_ is a circumferential size of a single cell, *N*).

The equation was then rewritten as follows:

(6) $$\begin{array}{c} sin\left(u\right)sin\left(v\right)sin\left(w\right)+sin\left(u\right)cos\left(v\right)cos\left(w\right)+cos\left(u\right)sin\left(v\right)cos\left(w\right)\\ +cos\left(u\right)cos\left(v\right)sin\left(w\right)=C \end{array}$$

wherein $u=\frac{2\pi U}{L_{U}}$; $v=\frac{2\pi V}{L_{V}}$; and $w=\frac{2\pi W}{N}$.

The following applies to the Diamond TPMS-based structure in the cylinder:

- Cell Radius (*L*_U_) is the cell size in the radial direction; if the L_U_ is enlarged, the cells will be larger and the TPMS-based structure will be less dense; if the L_U_ is reduced, the cells will be smaller and the structure will be denser.
- Cell Height (*L*_V_) is the cell size along the cylinder axis; if the L_V_ is enlarged, the pattern will be stretched and the cells will be higher.
- Arc Count (*N*) is the number of cells around the perimeter and expresses how many times the TPMS will occur repeatedly around the cylinder’s perimeter.
- Thickness (t) is the thickness of the material constituting the TPMS walls; a TPMS with zero thickness is first formed, and only then is the material added on both sides.

In this paper, the effects of the individual parameters on the surface area and the volume of the solid structure were analysed, while three experimental series were conducted:

- A change in the cell count around the perimeter (*N*) with a maintained cell size (U, V) and a maintained wall thickness range (*t*_min–tmax_);
- A change in the cell size (*U*, *V*) with a maintained cell count (*N*) and a maintained wall thickness range (*t*_min_–*t*_max_);
- A change in the wall thickness range (*t*_min_–*t*_max_) with a maintained cell count (*N*) and a maintained cell size (*U*, *V*).

In this analysis (Table 2), the effect of the cell count around the perimeter (Arc Count) on the surface area and the volume of the TPMS-based structure was analysed, with a maintained wall thickness (Thickness 1.8–2.2 mm) and three different cell sizes (Cell Radius—65, 55, and 45 mm; Cell Height—50, 40, and 30 mm).

**Figure 3:**
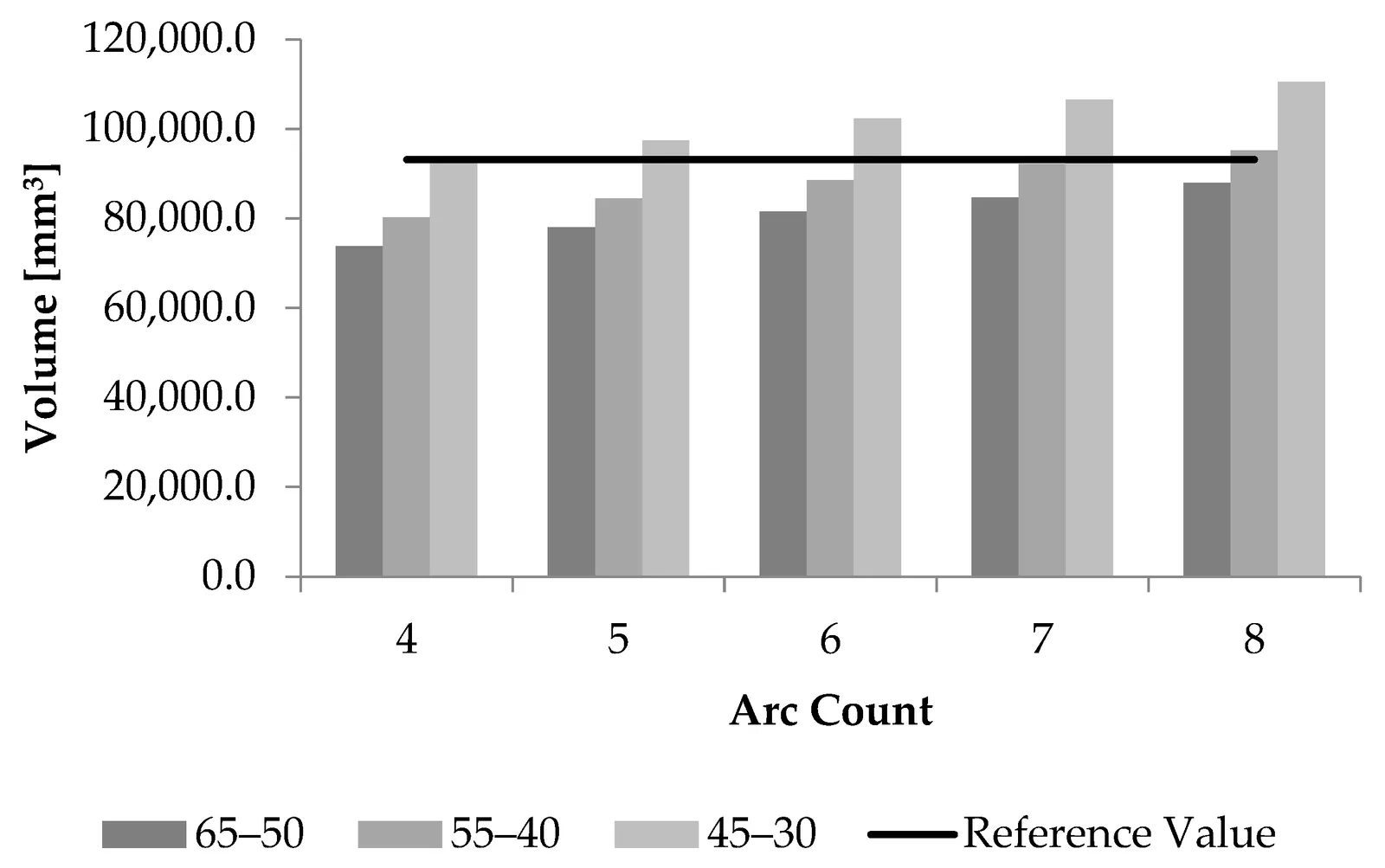
Volume of the TPMS-based structure with a changed cell count around the perimeter.

Results showed that with an increasing Arc Count value, the volume of the structure also increased for all of the cell sizes (Figure 3). An increase in Arc Count from 4 to 8 caused an increase in the surface area and volume, while the most significant increase was observed for the smallest cell size—Cell 45–30. A comparison of the individual cell sizes clearly showed that the smaller cell sizes led to higher values of surface area and volume with the same Arc Count value. For example, at Arc Count = 7, the structure of Cell 45–30 exhibited a surface area of 164,085.2 mm^2^ and a volume of 106,586.4 mm^3^, whereas the values for Cell 65–50 were lower, in particular, a surface area of 130,932.4 mm^2^ and a volume of 84,680.5 mm^3^. The highest values were achieved with a combination of Cell 45–30 and Arc Count = 8; on the other hand, the lowest values were observed with a combination of Cell 65–50 and Arc Count = 4. As the cell size decreased, the structure was denser since more cells fitted into the same space. Compared to the reference volume value, the most appropriate configuration was apparently Cell 55–40 at Arc Count = 7 since the related volume amounted to 92,210.3 mm^3^ and the volume fraction was 99.02%.

In the second series of experiments, the effect of the cell size on the surface area and volume of the TPMS-based structure was analysed, while the cell count (*N*—2, 4, 7) and the wall thickness range (thickness 1.8–2.2 mm) remained unchanged (Table 3).

**Figure 4:**
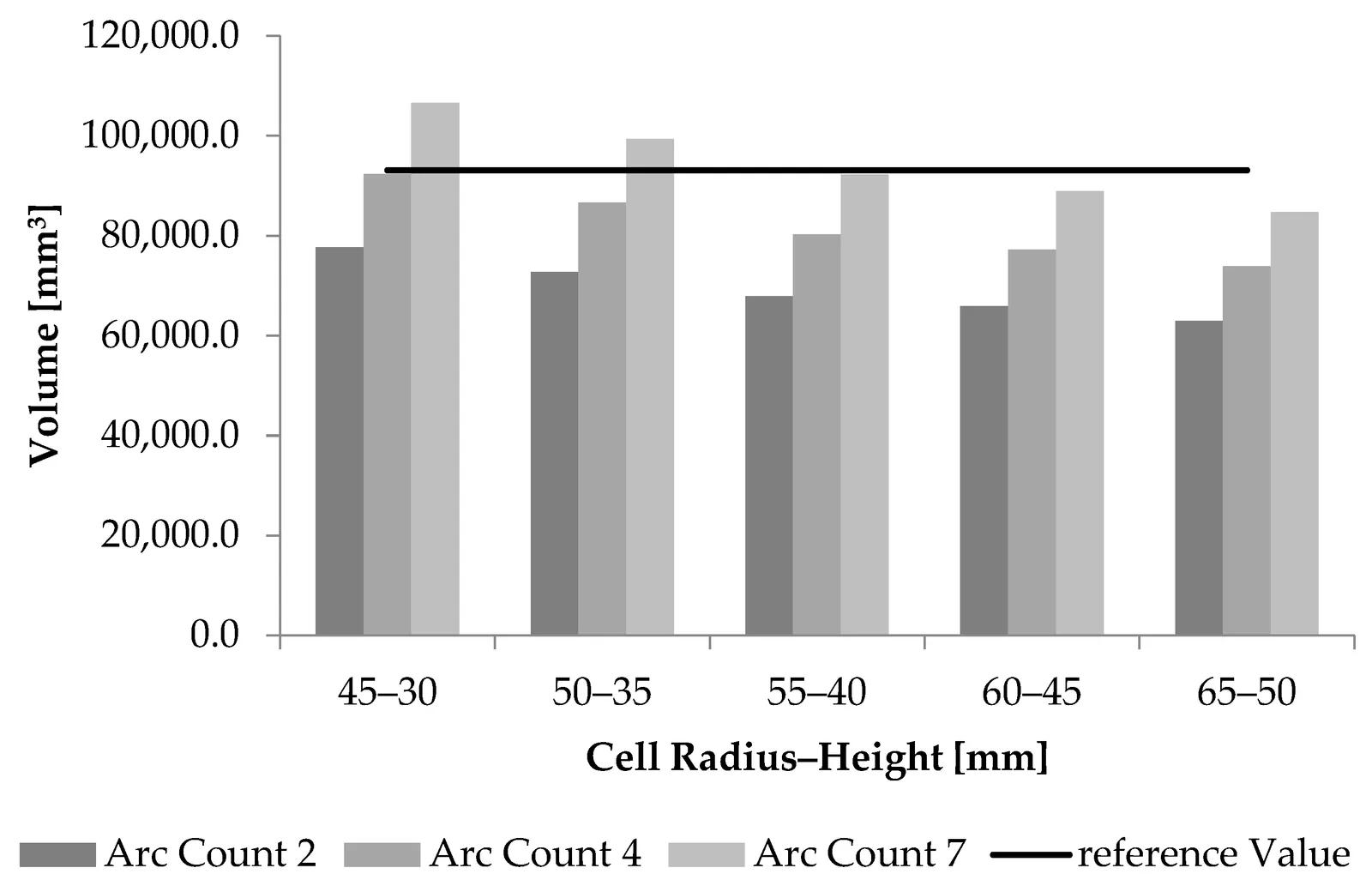
Volume of the TPMS-based structure with a changed cell size.

Results showed that a change in the cell size (Cell Radius and Cell Height) significantly affected the surface area, volume, and volume fraction of the TPMS-based structure for all of the Arc Count values. As the cell size increased from 45–30 mm to 65–50 mm, the surface area and volume of the structure gradually decreased. This trend was observed for all of the Arc Count configurations. By contrast, as the Arc Count increased, the surface area and volume of the structure increased. The highest values were observed with Arc Count = 7; the structure exhibited the largest surface area and the highest volume in all of the cases. For example, with a cell size of 45–30 mm, the volume amounted to 106,586.4 mm^3^, whereas with the same configuration and Arc Count = 2, the volume value was as low as 77,657.5 mm^3^. Compared to the reference volume value, the most appropriate configuration was as follows: Cell Radius = 55 mm; Cell Height = 40 mm; and Arc Count = 7 (Figure 4). With this configuration, in which the volume amounted to 92,210.3 mm^3^ and the volume fraction was 99.02%, the resulting value came closest to the required reference value and the surface area was larger than the values achieved with lower Arc Count values.

In the third series of experiments, the effect of variations in the wall thickness (t_min_–t_max_) on the geometric parameters of the TPMS-based structure was analysed, while the cell count (*N*) and cell size (*U*, *V*) remained unchanged. The analysis was carried out for *N* = 4 and *N* = 7 and for the cell sizes Cell 45–30 mm and Cell 65–50 mm (Table 4).

**Figure 5:**
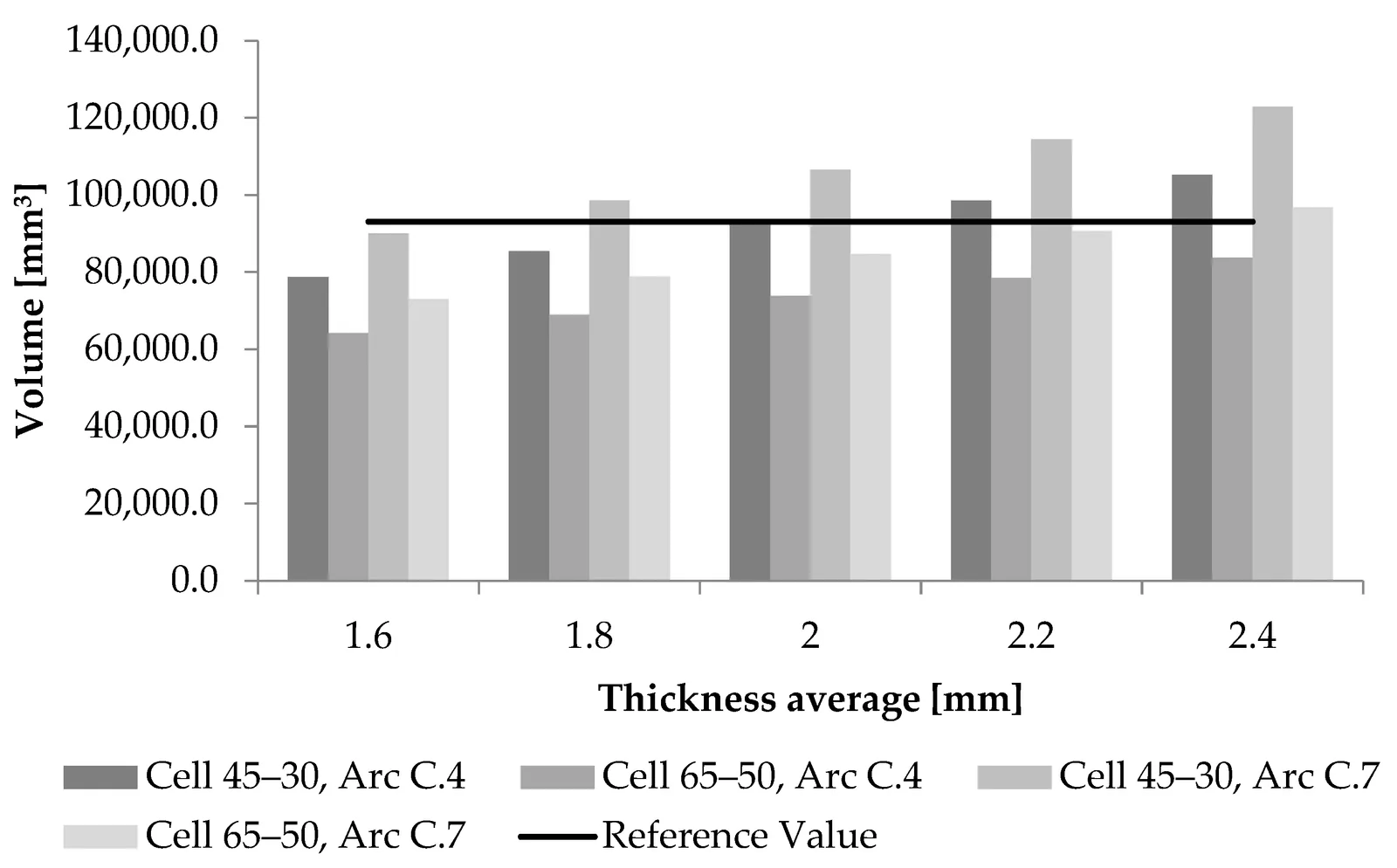
Volume of the TPMS-based structure with a changed wall thickness.

Results showed that with an increasing wall thickness, the volume of the structure and the volume fraction gradually increased in all of the analysed configurations (Figure 5). An increase in the wall thickness from 1.4–1.8 mm to 2.2–2.6 mm resulted in an increase in volume with all of the applied parameter combinations. The highest increase was observed with the Cell 45–30 mm and Arc Count = 7 configuration, in which the volume increased from 90,059.9 mm^3^ (at a wall thickness of 1.4–1.8 mm) to 122,939.0 mm^3^ (at a wall thickness of 1.4–1.8 mm). A comparison of the cell size effects revealed that with a lower cell size (Cell 45–30 mm) and the same wall thickness value, the volume and volume fraction values were higher than those achieved with a larger cell size (Cell 65–50 mm). The same trend was observed for both Arc Count values. When the Arc Count increased from 4 to 7, the volume of the structure increased, while the highest values were achieved with a combination of Cell 45–30 mm, Arc Count = 7, and a wall thickness of 2.2–2.6 mm. In order to achieve the volume closest to the reference value, the most appropriate configuration is Cell 45–30 mm, Arc Count = 4, and a wall thickness of 1.8–2.2 mm, in which the volume fraction amounted to 99.14%.

Based on the conducted analyses, it may be stated that the geometric parameters of the TPMS-based structure significantly affected the resulting surface area, volume, and density of the structure. An increase in the Arc Count parameter led to a higher complexity of the structure, which was manifested in elevated values of the surface area, volume, and volume fraction. By contrast, an increase in the cell dimension resulted in a decrease in the total surface area and volume of the structure, while the smaller cell units were associated with higher density of the structure and higher surface area values with a maintained cell count. A change in the wall thickness had a direct effect on the volume of the TPMS-based structure; as the wall thickness increased, the volume and volume fraction values also increased. This effect was more significant with the smaller cell size and higher values of the Arc Count parameter.

Results confirmed that with an optimal combination of the cell size (*U*, *V*—Cell Radius–Height), cell count around the perimeter (*N*—Arc Count), and wall thickness (t_min_–t_max_) parameters, geometric properties of the TPMS-based structure may be intentionally affected and adjusted to any required volume and surface area characteristics.

**Figure 6:**
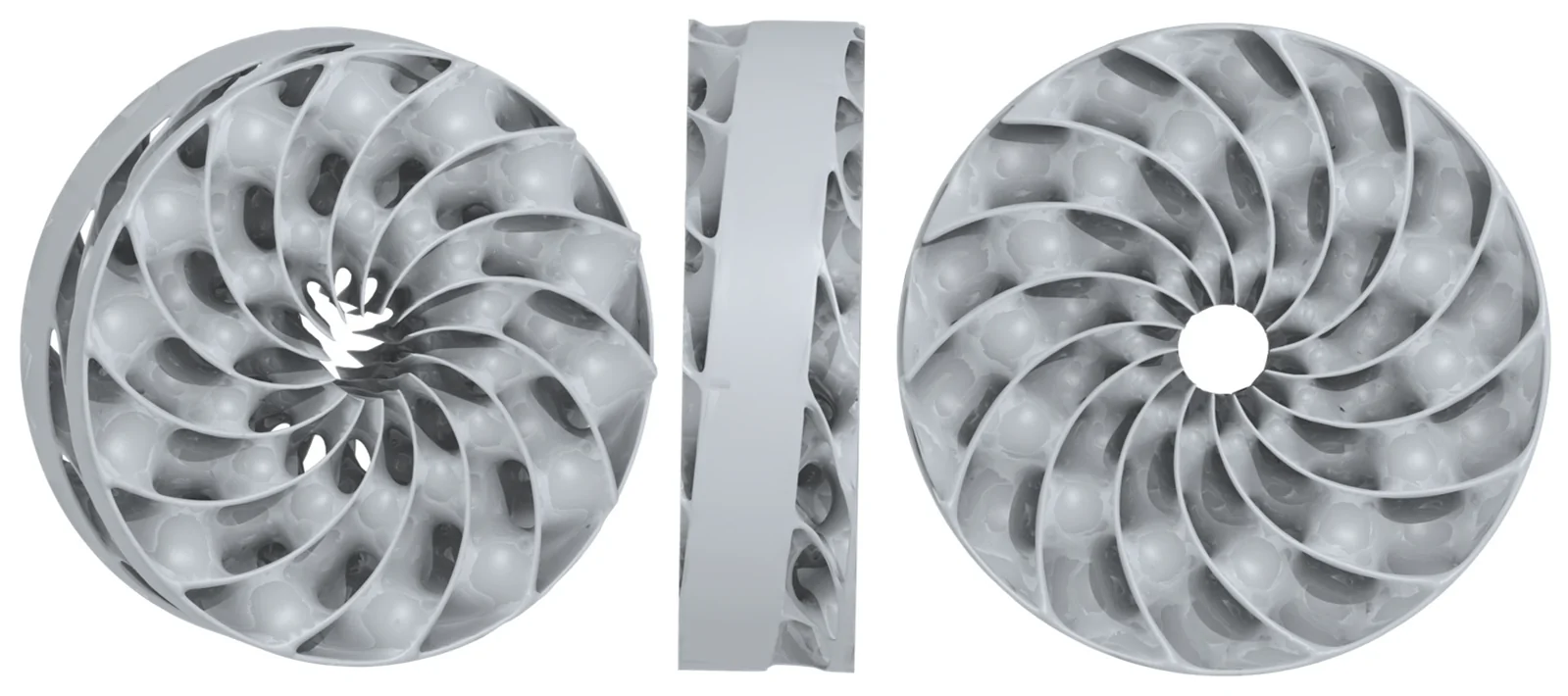
Image of the resulting geometry of the passive heat exchanger created using the TPMS (a 40 mm long section).

The TPMS-based structure with the most suitable configuration was the one with the following parameters: Cell 55–40 mm; Arc Count = 7; and a wall thickness range of 1.8–2.2 mm. This configuration was assessed as the optimal one since the achieved volume fraction was 99.02% compared to the one achieved with the original geometry of the internal heat exchanger in the certified MNTZV-159 storage tank (the reference value). This ensures the same volume of the exchanger, and hence, also of the alloy that will be placed in the storage tank. Moreover, this configuration facilitated achieving a higher value of the surface area than the values achieved with the configurations with lower Arc Count values, while the density of the TPMS-based structure was maintained at an appropriate level. The structure density also determines the maximum distance of MH particles from the exchanger surface. Despite the fact that the TPMS structure with a cell parameter of 45–30 mm, an Arc Count of 4, and a wall thickness range of 1.8–2.2 mm exhibited a volume fraction of 99.14% (closer to the reference sample), the structure selected for this analysis exhibited a shorter maximum distance between MH particles and the exchanger surface. Moreover, the selected configuration of Cell 55–40 mm and Arc Count = 7 facilitated easier vertical filling of the storage tank with a powder alloy prior to the activation. The resulting geometry of the passive heat exchanger created using the TPMS is shown in Figure 6.

The volume fraction value was exceeded primarily in cases with higher Arc Count values and lower cell size values. Such combinations resulted in higher complexity and density of the structure. Therefore, in order to select an optimal model, it is advisable to choose the values approaching 100%, not the highest values of the volume fraction. For example, for Cell 45–30, Arc Count 5 → 104.60% means that the volume is approximately 4.6% higher than the reference value.

## 3. Numerical Calculation of Heat Transport

The enhancement of hydrogen absorption kinetics depended on the ability of the MH storage system to remove the generated heat from the metal hydride volume. Increasing the effective thermal conductivity facilitated a reduction in the average temperature of the MH bed; according to Van ’t Hoff‘s law, this also reduces the equilibrium pressure. Reaction kinetics depend on the ratio of hydrogen pressure to equilibrium pressure; therefore, the heat-transfer intensification must also result in faster hydrogen absorption at the same gas pressure. Also, according to the Arrhenius equation, a decrease in reaction temperature also increases the reaction kinetics of hydrogen absorption. Based on the aforementioned reasons, increasing the efficiency of heat transfer in the MH bed is proportional to the absorption enhancement and the reduction in the time of tank refuelling with hydrogen [28].

**Figure 7:**
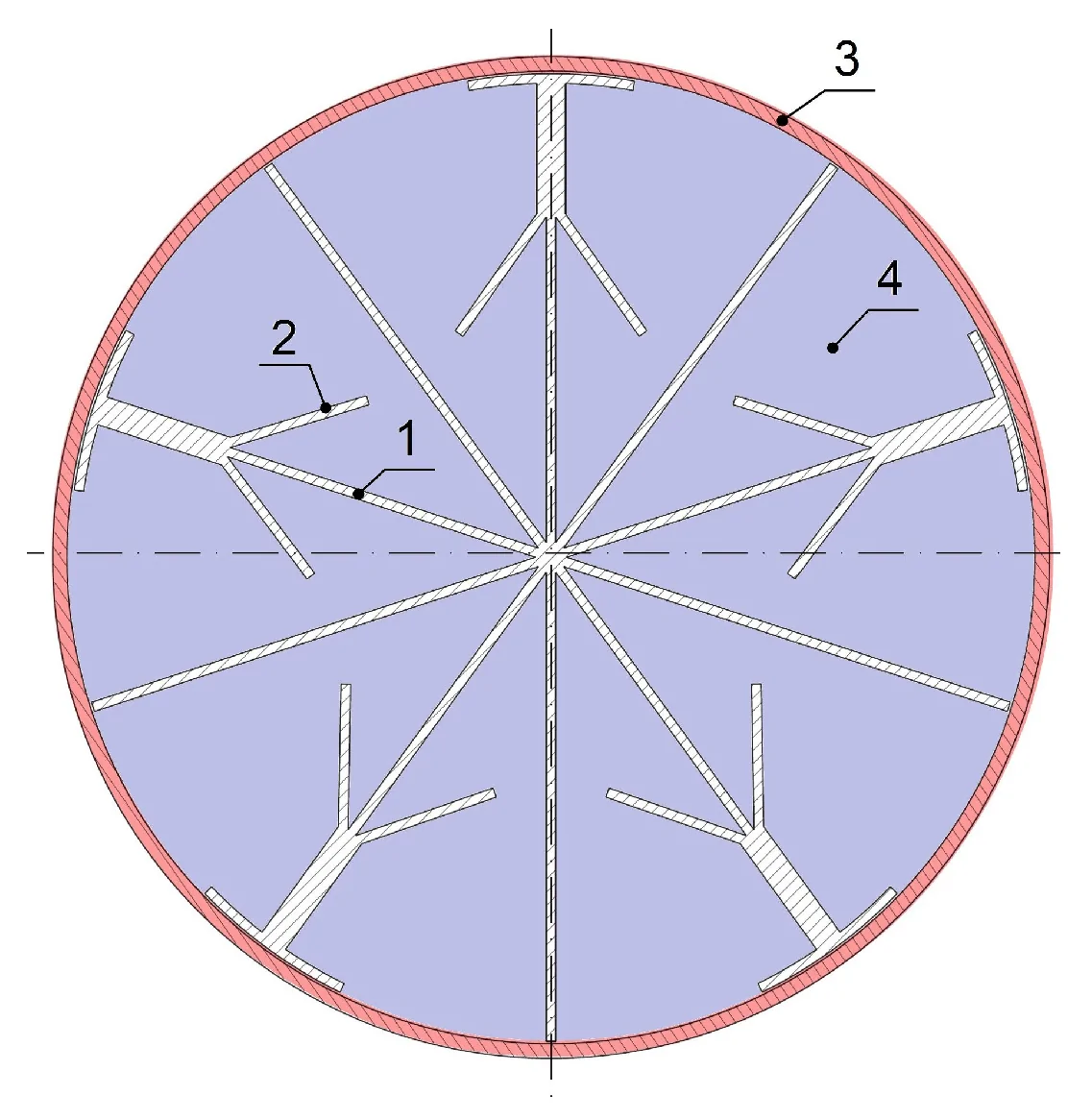
Arrangement of the MNTZV-159 storage tank in a cross-section: 1—primary fins; 2—secondary fins; 3—external shell; 4—powder metal hydride in the space between the fins.

The numerical calculation was made with the aim of comparing the ability to remove heat from the bed of the metal hydride material. The comparison was made with the reference sample and the TPMS-based structure with the following parameters: Cell 55–40 mm; Arc Count = 7; and a wall thickness range of 1.8–2.2 mm. The spatio-temporal distribution of temperature in the volume of the metal hydride bed was analysed through a numerical calculation that was made in the ANSYS CFX software environment (2024R1, ANSYS Inc., Canonsburg, PA, USA). Two different modifications of the internal heat exchanger with a diameter of 148 mm were used. The first one was the geometry of a certified MNTZV-159 storage tank (Figure 2) and the second one was the geometry created using TPMSs (Figure 6). The numerical calculation was made as a steady-state calculation, with the same internal heat source and a shell cooling system. The heat exchanger geometry was 40 mm long (a cut from the storage tank), which corresponded to the periodicity of the repeating TPMS geometric pattern. The arrangement of the individual domains is shown in Figure 7. The aluminium heat exchanger was installed in the external shell made of stainless steel with an inner diameter of 150 mm and a wall thickness of 4.5 mm. The free space between the fins was filled with a powder MH alloy.

Volumes of the individual domains are listed in Table 5. The volumes of the material were similar for the heat exchanger and for the metal hydride bed, with a deviation of 0.98%.

**Figure 8:**
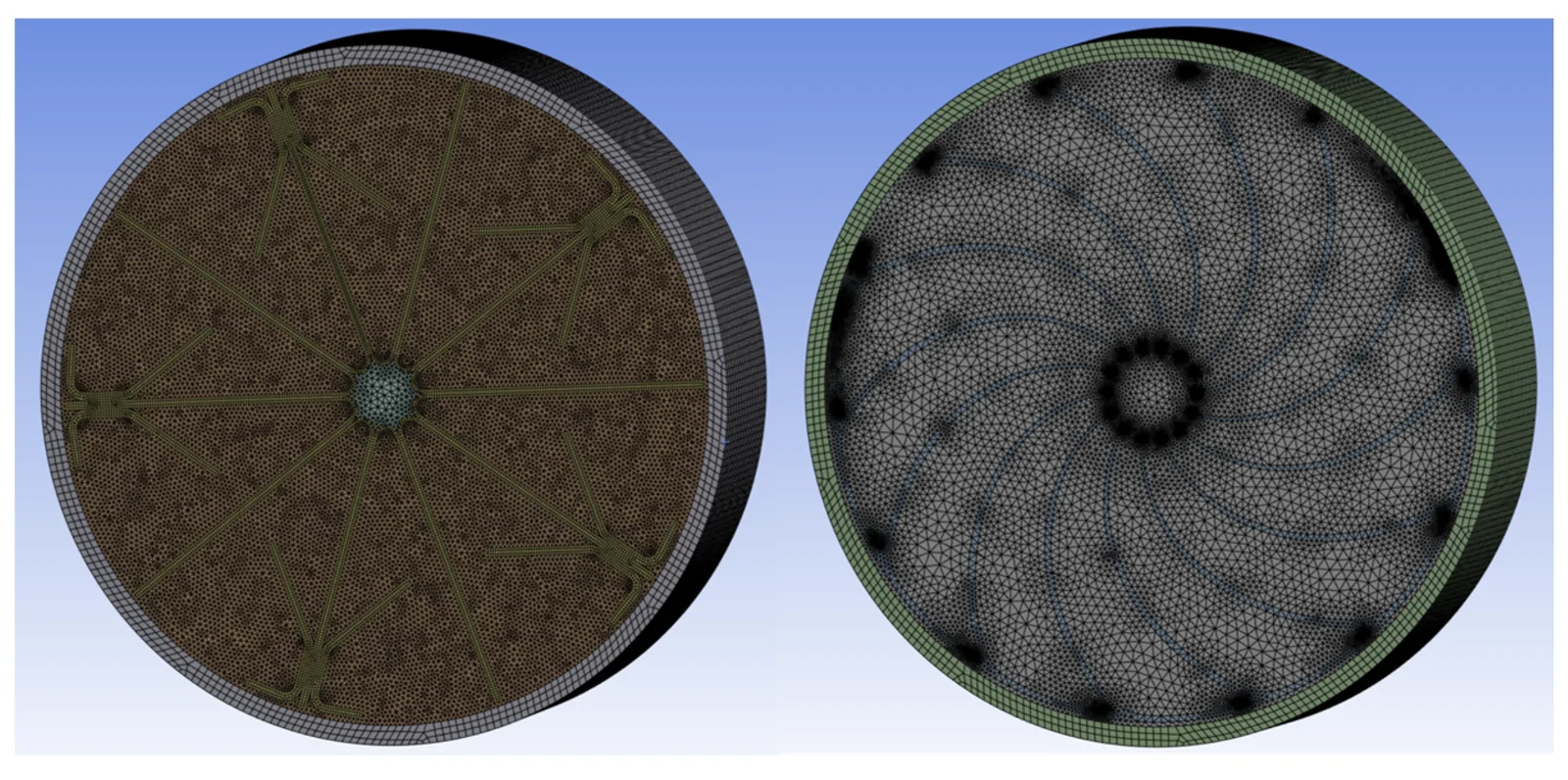
Image of the meshes of the individual variants.

For the purpose of discretisation of the domains of the subsequent numerical calculation of heat transport, an unstructured tetrahedral mesh was used (Figure 8). Total counts of the mesh elements are listed in Table 6.

The MNTZV-159 geometry was subjected to a sensitivity analysis for mesh fineness; it showed that as the metal hydride mesh fineness increased, the maximum temperature of metal hydride gradually decreased. This was caused by the low thermal conductivity of the domain, while the smaller mesh elements facilitated a more accurate calculation of heat transfer. When the metal hydride mesh element count exceeded 5 million, the calculated thermal field was rather stable and exhibited only minimal changes (Table 7). Due to a larger curvature and thinner elements, the TPMS geometry required a mesh denser than that of the MNTZV-159 (Table 8).

The size of the narrow zones in the mesh of the MH material on the border between the heat exchanger and the external shell was less than 0.5 mm in order to ensure sufficient resolution and calculation accuracy. The average skewness of the mesh amounted to 0.24 for the TPMS and 0.20 for the MNTZV-159.

In order to isolate the dominant heat transport mechanisms, the model was defined based on several simplifying assumptions. The exothermic nature of hydrogen absorption was represented by a homogeneously distributed isotropic volumetric heat source in the metal hydride alloy. The effect of the flow of gaseous hydrogen in the porous structure of the bed was not taken into consideration, nor were the changes in the equilibrium pressure, as described by the Van ’t Hoff equation. Throughout the simulation, the thermophysical properties of all the modelled materials, in particular thermal conductivity and specific heat capacity, were regarded as constant and unaffected by temperature (Table 9).

Taking into account the aforesaid assumptions, the heat transport in the individual solid domains was expressed using a general differential equation for heat transfer with a volumetric energy source:

(7) $$0=\alpha \cdot \nabla^{2}T+\frac{q_{is}}{\rho \cdot c}\;(K\cdot s^{-1})$$

wherein α is the thermal diffusivity of the material (m^2^∙s^−1^); ∇*T* is the temperature gradient in the Cartesian coordinate system (K∙m^−1^); *q*_is_ is the thermal power generated or absorbed in a volume unit (W∙m^−3^); *ρ* is the density of the analysed material (kg∙m^−3^); and *c* is the heat capacity (J∙kg^−1^∙K^−1^) [25].

Numerical simulations were carried out for a metal hydride alloy with a maximum hydrogen storage capacity of 1.8 wt%. The task was carried out as time-independent, and a constant internal heat source with a heat output of 333.74 W was used; in mathematical terms, this corresponded to the average heat output generated in the 40 mm long section of the storage tank at a hydrogen flow rate of 0.27·10^−3^ m^3^·s^−1^.

(8) $$P=Q_{V-H2}\cdot q_{rh}\;(W)$$

wherein *Q*_V-H2_ is the flow rate of hydrogen flowing into the 40 mm long section of the storage tank (m^3^·s^−1^) and *q*_rh_ is the reaction heat of the MH alloy (J·m^−3^).

The aforementioned flow rate corresponds to the average hydrogen flow rate that would theoretically increase the capacity of Hydralloy C5 in that section of the storage tank from zero to 1.32 wt% in 20 min (refuelling time), as calculated using the following equation:

(9) $$Q_{V-H2}=\frac{c\;\cdot \;\rho_{MH\;}\cdot {\;V}_{MH\;}}{\rho_{H2\;}\cdot \Delta \tau }\cdot q_{rh}\;(m^{3}\cdot s^{-1})\;$$

wherein *c* is the capacity of absorbed hydrogen (1); $\rho_{MH}$ is the bulk density of metal hydride (kg·m^−3^); *V*_MH_ is the metal hydride volume (m^3^); $\rho_{H2}$ is the hydrogen density in normal conditions (kg·m^−3^); and ∆τ is the time interval until the capacity *c* is achieved (s).

On the interface between the storage tank surface and the surrounding environment, a boundary condition—convective heat transfer—was defined; it was expressed through the equality of convective and conductive heat fluxes. This formulation facilitated taking into account the heat removal from the storage tank to the surrounding environment while maintaining the physical consistency of the model.

(10) $$-k\frac{\partial T}{\partial n}=h_{amb}\cdot \left(T_{w}-T_{amb}\right)\;(W\cdot m^{-2})\;$$

wherein *k* is the thermal conductivity of the steel tank (W·m^−1^·K^−1^); *n* is the distance in the direction of the normal line to the interface area (m); *h*_amb_ is the overall heat-transfer coefficient for the area between the tank surface and the surrounding environment (W·m^−2^·K^−1^); *T*_w_ is the temperature of the surface of the wall of the MH tank (K); and *T*_amb_ is the ambient temperature (K).

The overall heat-transfer coefficient value that was used in the numerical calculation was based on the values of the certified MNTZV-159 storage tank identified by measurements, and it amounted to 967 W·m^−2^·K^−1^, while the cooling water temperature was 20 °C. Based on the calculations made using critical equations for heat transfer, the aforesaid value of the overall heat-transfer coefficient corresponds to the average flow rate of water on the surface of the shell (0.25 m·s^−1^).

The boundary condition of symmetry was applied to both axial surfaces; this means that the storage tank taken into consideration was long, while the thermal impact of the terminal elliptical parts was neglected. The internal heat exchanger was in contact with the steel shell through the 1 mm thick MH layer, while the interface flux was set as conservative.

## 4. Results and Discussion

Results of the process of designing the passive heat exchanger indicated that the increasing Arc Count value in the software tool increased the complexity of the structure and, consequently, led to elevated values of surface area and volume, whereas the reduction in the cell size led to a higher density of the TPMS structure as well as higher values of surface area and volume with the same cell count. The results therefore showed that increasing the Arc Count parameter led to a higher density and surface area of the TPMS structure, whereas increasing the cell size resulted in a reduced total surface area and volume of the structure. The results also showed that with increasing wall thickness, the volume of the TPMS structure also increased, while this effect was more notable when the cell size was smaller and the Arc Count value was higher.

The newly designed TPMS-based structure was compared to the heat exchanger of the certified MNTZV-159 storage tank through a numerical calculation of heat transfer during hydrogen absorption. The internal heat output values were identical in both geometries, based on a comparison of the maximum and average temperatures in the volume of the metal hydride bed. The concept of the new design was based on the assumption that an increase in the effective thermal conductivity of the active volume of the storage tank would lead to a reduction in temperature gradients formed during the exothermic reaction of hydrogen absorption. This effect was achieved by the integration of an aluminium structure with a significantly higher thermal conductivity compared to that of the metal hydride alloy, and this supported the transport of reaction heat from the central part of the bed towards the shell heat exchanger. Moreover, homogeneity of the thermal field increased throughout the storage tank volume.

**Figure 9:**
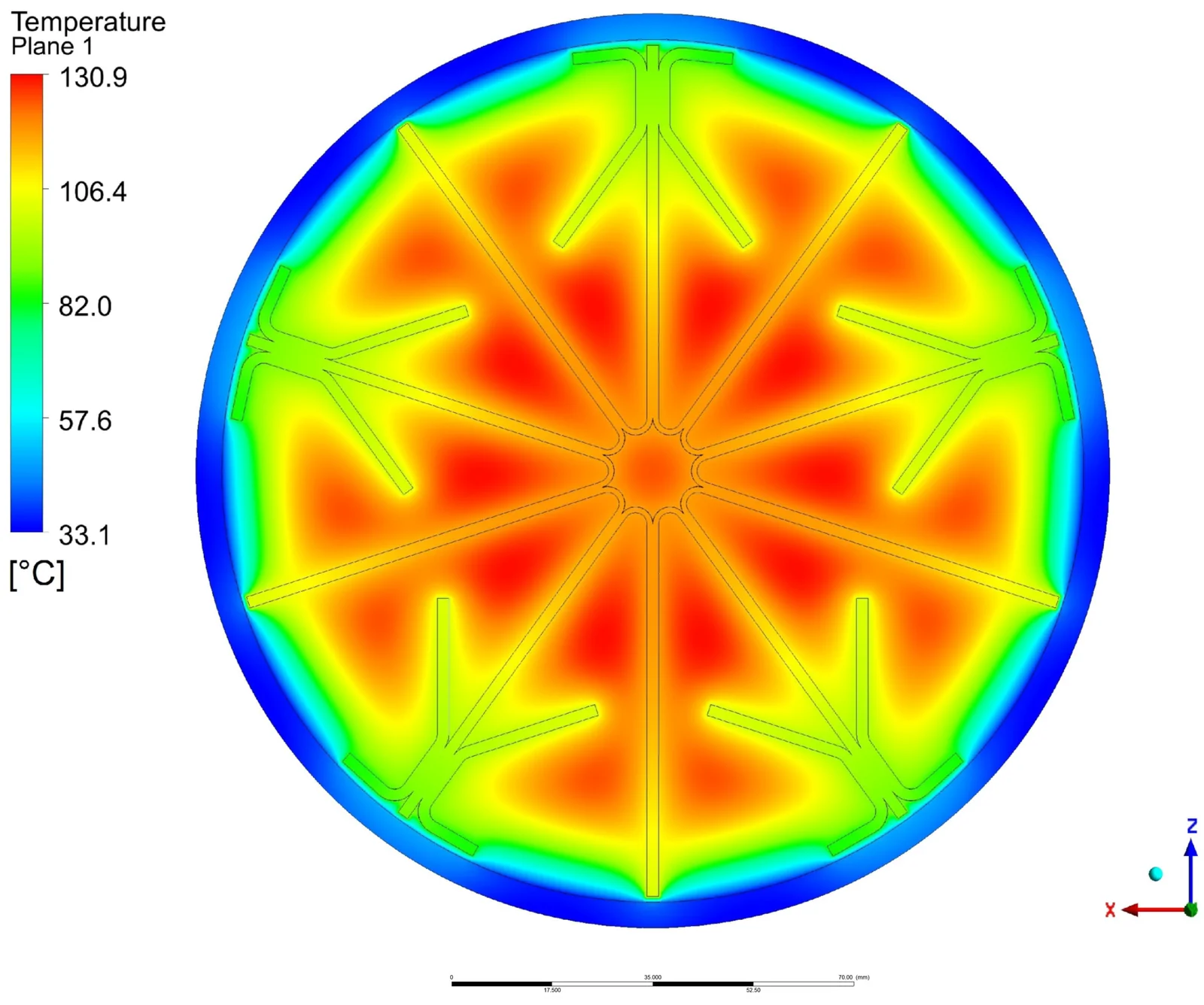
Thermal field in a cross-section of the reference domain of the MNTZV-159.

**Figure 10:**
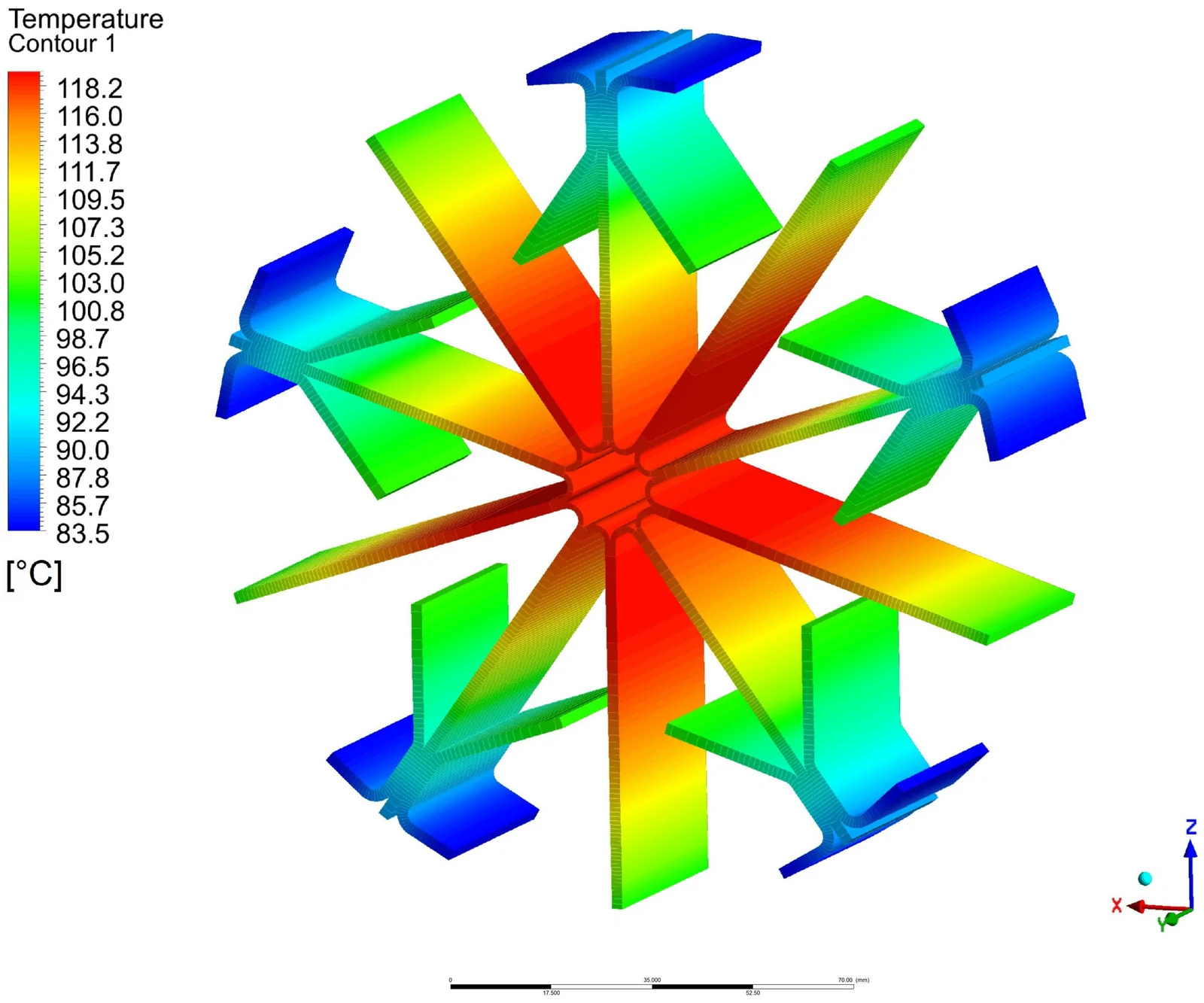
Thermal field on the surface of the MNTZV-159 heat exchanger.

The numerically calculated thermal field of the domain with the MNTZV-159 heat exchanger is shown in Figure 9. Hydrogen absorption into the alloy structure resulted in the generation of heat, which increased the total temperature of the metal hydride. Heat was transported through the metal hydride and the heat exchanger fins towards the shell of the storage tank; subsequently, it was removed from that area with cooling fluid. The improvement in heat removal and the homogenisation of the thermal field was facilitated by the use of the primary and secondary fins of the heat exchanger. Their temperature gradually decreased in the radial direction, and this created a temperature gradient that facilitated heat transport from the core of the storage tank towards the shell (Figure 10). Local thermal field peaks were formed in the volume of metal hydride between the primary and secondary fins.

**Figure 11:**
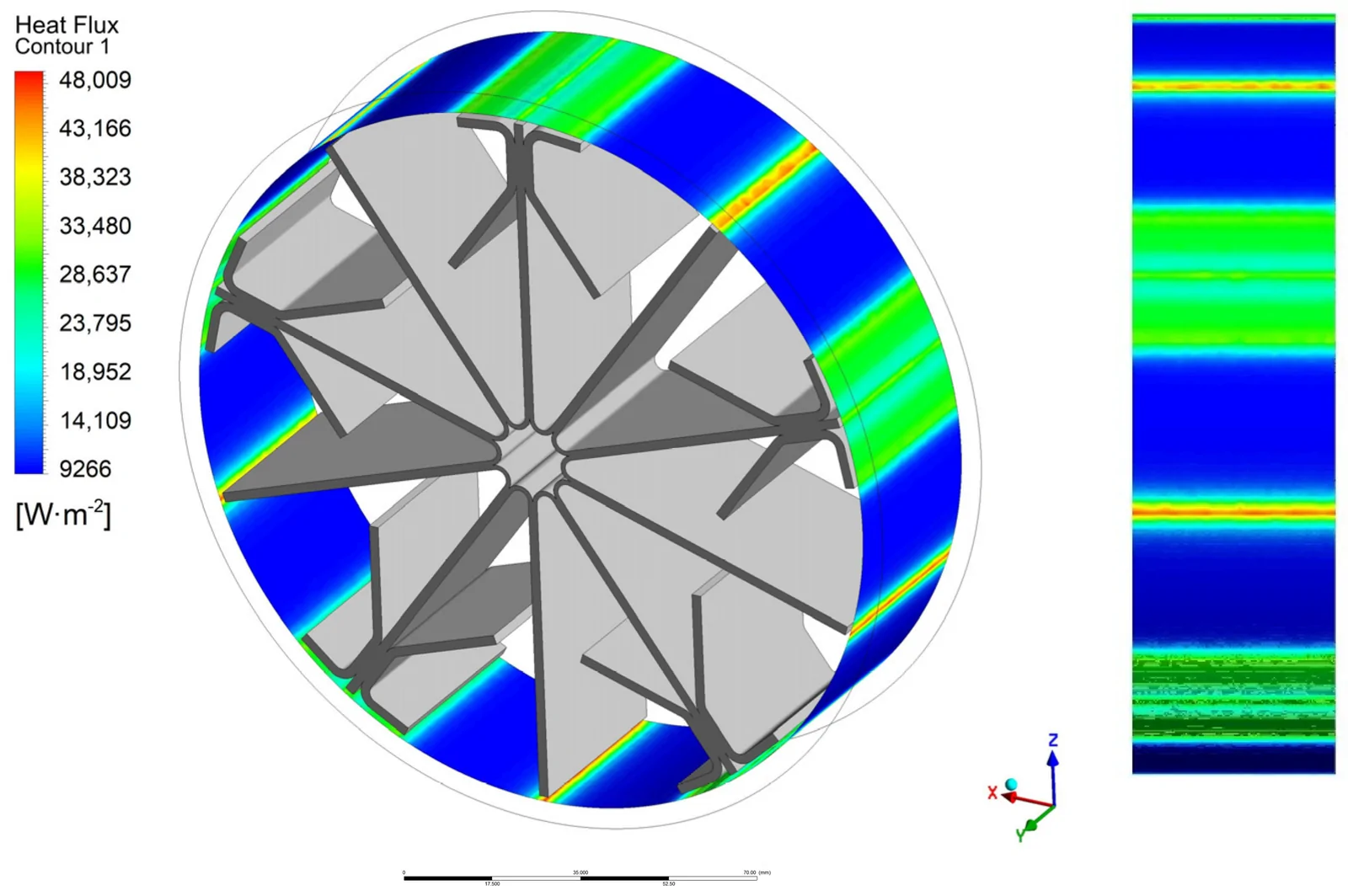
Heat flux on the inner radius of the steel shell of the MNTZV-159 heat exchanger.

In the immediate vicinity of the outer metal shell, there were zones with elevated heat flux density, which was caused by heat transport through the primary and secondary fins of the heat exchanger. This heat flux, recalculated for a unit surface area, amounted to a value that was 3–5 times higher than the value of heat flux density in the zones with a higher volume of metal hydride (blue surfaces in Figure 11).

**Figure 12:**
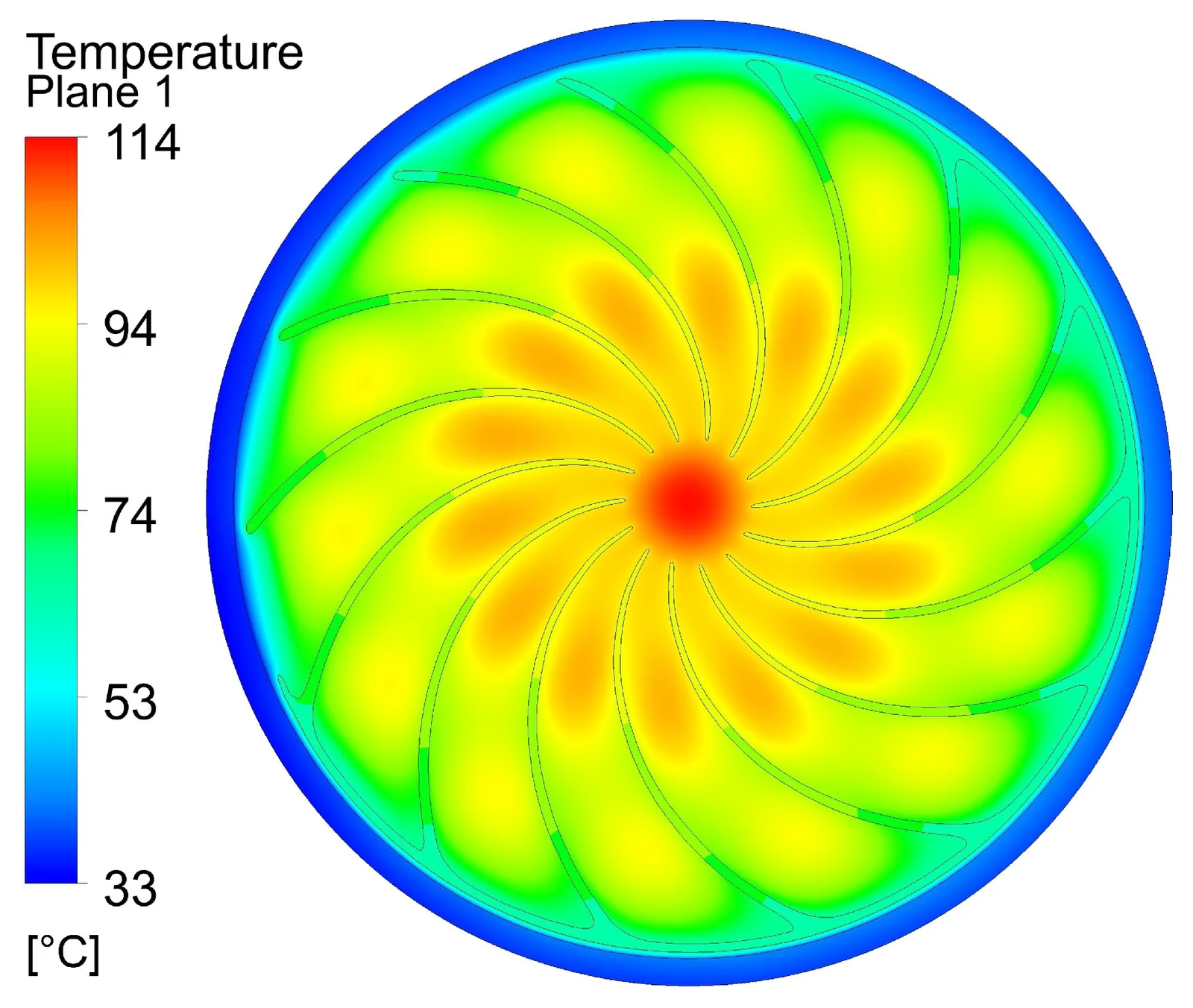
Thermal field in a cross-section of the domain with the TPMS heat exchanger.

The thermal field in a cross-section of the TPMS domain exhibited a decrease in the maximum temperature of metal hydride, with a local peak located in the axis of the storage tank. Due to the use of the heat exchanger with Triply Periodic Minimal Surfaces (Figure 12), the thermal field was homogenised, and hence, the temperature differences in the volume of metal hydride were eliminated. This increases the probability of accelerated hydrogen absorption, or reduced hydrogen pressure, compared to those of the MNTZV-159 heat exchanger, as calculated using Equation (2).

**Figure 13:**
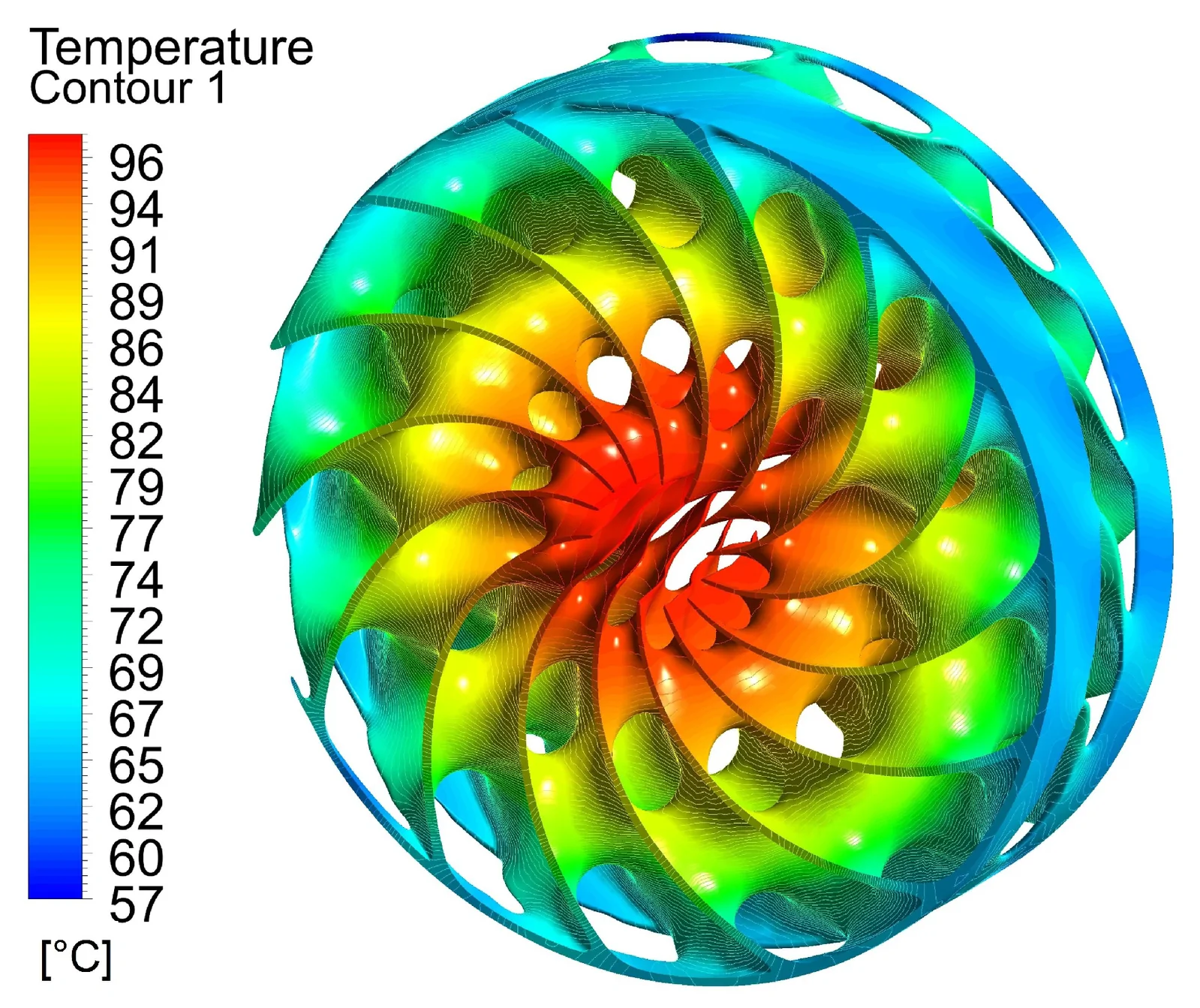
Thermal field on the surface of the TPMS heat exchanger.

Temperatures on the surface of the TPMS heat exchanger are shown in Figure 13. Compared to the thermal field of the MNTZV-159, the temperatures are significantly shifted towards lower values, and this facilitated the improvement of heat transfer between metal hydride and the heat exchanger. This temperature decrease was caused by the reduction in the average distance between the surface of the internal heat exchanger and powder metal hydride particles as a result of elevated surface temperature and an optimal surface curvature. The result was an elevated temperature gradient, which, according to Fourier’s law of heat conduction, increases the local heat flux.

**Figure 14:**
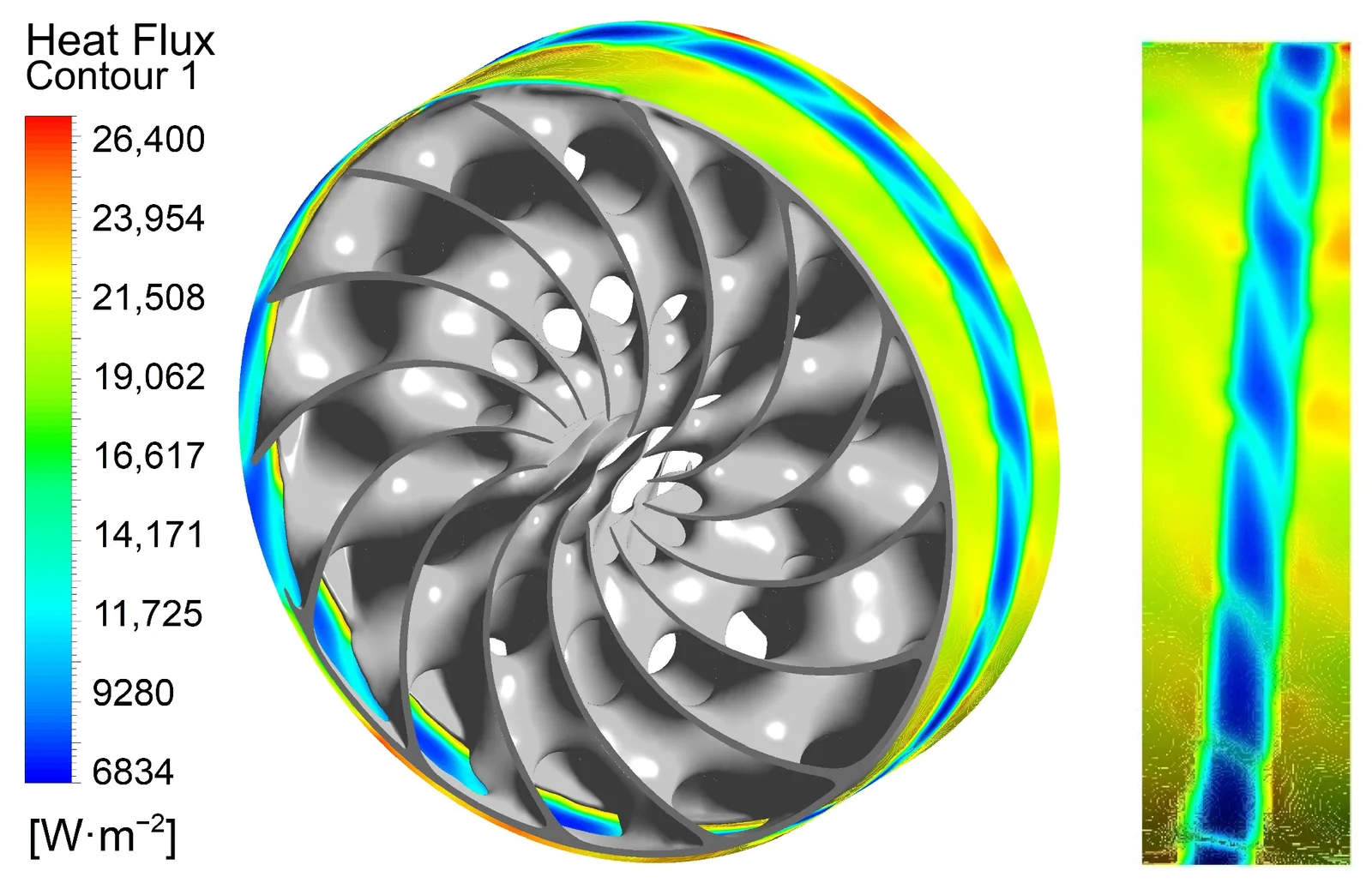
Heat flux on the inner radius of the steel shell of the TPMS heat exchanger.

Values of heat flux density near the outer metal shell were lower than those observed with the reference heat exchanger. However, it was distributed more evenly on the outer heat-transfer surface (Figure 14).

In both analysed geometries, the heat fluxes generated in metal hydride were identical; therefore, the heat fluxes removed to the shell cooling system were also identical. The main added value of the utilisation of the TPMS structures was the reduction in local temperature peaks as well as the total average temperature of metal hydride, as shown in Table 10.

The use of the TPMS heat exchanger, compared to the use of the MNTZV heat exchanger, resulted in a 15.24% decrease in the difference between the maximum metal hydride temperature and ambient temperature. When comparing the difference between the average metal hydride temperature and ambient temperature, the decrease amounted to as much as 24.52%. A decrease in the average temperature from 107.7 °C to 86.2 °C facilitated the reduction in the calculated equilibrium pressure of the used Hydralloy C5 from 9.86 MPa to 6.42 MPa, as calculated using Equation (2). At constant generation of heat in a numerical simulation in a steady-state calculation, the achieved hypothetical pressures would exceed the maximum allowable pressure in the certified tank. Therefore, the utilisation of the tank would require a reduction in the input flow rate in order to prevent exceeding the pressure. With regard to the demonstrated reduction in the average theoretical temperature of MH, the use of TPMS is likely to reduce the operational pressure of MH and facilitate achieving a shorter time of hydrogen absorption also in storage tanks used in real-life applications.

## 5. Conclusions

The presented study investigated the use of Triply Periodic Minimal Surface (TPMS) structures as a passive internal heat exchanger for the certified MNTZV-159 low-pressure metal hydride hydrogen storage tank. The research combined the parametric geometric improvement with a numerical analysis of heat transfer with the aim of identifying a configuration capable of improving the thermal management system while maintaining the original storage capacity. Compared with conventional finned heat exchangers, TPMS structures provide a highly interconnected three-dimensional geometry with a high surface-to-volume ratio, hence promoting more effective heat conduction through the metal hydride bed.

The geometric analysis confirmed that all of the investigated design parameters significantly affected the final TPMS characteristics. Increasing the Arc Count resulted in an increase in both surface area and solid volume, whereas larger cell dimensions led to a reduction in those values due to a lower structural density. Wall thickness mainly affected the solid volume, and this facilitated the accurate adjustment of the TPMS geometry to match the reference heat exchanger. The most suitable configuration was the one with Cell Radius = 55 mm, Cell Height = 40 mm, Arc Count = 7, and a wall thickness between 1.8 and 2.2 mm. With this design, a value of 99.02% of the reference heat exchanger volume was achieved. Also, nearly the same amount of active metal hydride was maintained while increasing the effective heat-transfer surface area. The fact that the volume of aluminium was almost identical means that the comparison reflected the effect of the geometry rather than an increase in the amount of conductive material.

Numerical simulations demonstrated that the replacement of a conventional finned heat exchanger with the newly designed TPMS-based structure substantially improved the thermal behaviour of the storage system. The average temperature of the metal hydride bed decreased from 107.7 °C to 86.2 °C, while the maximum temperature decreased from 130.9 °C to 114.0 °C. The difference between the maximum bed temperature and ambient temperature was reduced by 15.24%, and the difference between average temperatures decreased by 24.52%. In addition, the TPMS-based structure generated a more homogeneous temperature field and reduced the local hot spots that typically occur between the conventional fins.

The proposed design is also compatible with metal additive manufacturing, particularly selective laser melting, which enables the fabrication of complex TPMS geometries that are not feasible with the use of conventional manufacturing methods. However, the main disadvantage is still a higher manufacturing cost compared to that of the original heat exchanger.

The study was limited to steady-state heat transfer and geometric parametrisation. The numerical model did not include a hydrogen flow through the porous medium, transient absorption kinetics, temperature-dependent thermophysical properties, and thermomechanical effects during the repeated hydriding cycles. Future investigation should therefore involve transient simulations, including absorption kinetics, experimental validation of additively manufactured TPMS heat exchangers, and a comparison of alternative topologies, such as Gyroid, Schwarz P, and Neovius structures. Additional parametrisation considering mechanical strength, manufacturability, and pressure-drop characteristics may further improve passive thermal management systems in metal hydride hydrogen storage systems. Overall, the results confirmed that TPMS-based heat exchangers are a promising alternative to conventional finned heat exchangers, as they offer improved temperature uniformity while maintaining the original storage capacity.

## Figures and Tables

**Table 1:** Comparison with the closest TPMS and MNTZV-159 reactor studies.

Study	Topology	Reactor Geometry	Heat-Transfer Mechanism	Operation	Validation	Key Limitations
Lesmana and Aziz [14]	Gyroid	Purpose-built TPMS reactor	Internal TPMS conduction coupled to external convection	Natural and forced-convection cooling	A numerical model validated against the published experiments	Numerical/theoretical TPMS reactor concept; MH expansion neglected; constant porosity, permeability, and thermal conductivity assumed
Lesmana et al. [15]	Gyroid	Laboratory TPMS reactor with LaNi5	Internal gyroid heat-transfer surface connected to the cooled/heated boundary	Natural cooling and liquid cooling/heating	Direct experimental absorption and desorption tests	Laboratory-scale demonstrator; no comparison with a certified full-scale vessel or equal-volume retrofit
Jiang et al. [16]	Parametric TPMS	Solid-state reactor compared with a finned configuration	Coupled fluid flow, heat transfer, hydrogen reaction, and mechanical stress	Active thermal management/coolant flow	Numerical multi-physics model and design-of-experiments analysis	Primarily numerical; no full-scale experimental validation of the complete TPMS reactor reported
Baseline condition of MNTZV-159 [25,26]	Conventional aluminium amplifier	Certified double-shell tank	Radial conduction through the Al amplifier into a shell cooled with water	Active cooling; a water circuit	3D numerical calculation and fully fledged operational measurements	Limitations resulting from the approved certification criteria
This study	Diamond TPMS + external edging	Additional installation, limited to the certified MNTZV-159	Heat redistribution from the MH core towards the shell with the use of a passive heat exchanger	A passive heat exchanger; current geometric limitation	The same domain and the same volume of the material; numerical comparison	Numerical calculation and a comparison with the baseline model

**Table 2:** Effect of the Arc Count parameter on the surface area and volume of the TPMS-based structure.

Arc Count	Cell 65–50		
	Surface Area (mm^2^)	Volume (mm^3^)	Volume Fraction (%)
4	109,651.8	73,832.9	79.28
5	116,838.7	78,033.6	83.79
6	123,652.7	81,563.1	87.58
7	130,932.4	84,680.5	90.93
8	138,772.7	87,902.4	94.39
	Cell 55–40		
4	123,981.1	80,251.9	86.18
5	128,931.8	84,494.1	90.73
6	135,795.7	88,544.2	95.08
7	143,382.8	92,210.3	99.02
8	150,339.7	95,232.8	102.26
	Cell 45–30		
4	147,146.8	92,325.7	99.14
5	151,217.8	97,405.7	104.60
6	157,858.2	102,370.6	109.93
7	164,085.2	106,586.4	114.45
8	170,440.4	110,496.4	118.65

**Table 3:** Effect of the Cell Radius and Cell Height parameters on the surface area and volume of the TPMS-based structure.

Cell Radius Height (mm)	Arc Count 2		
	Surface Area (mm^2^)	Volume (mm^3^)	Volume Fraction (%)
45–30	138,379.9	77,657.5	83.39
50–35	126,542.0	72,702.9	78.07
55–40	113,834.7	67,838.3	72.85
60–45	108,357.9	65,886.8	70.75
65–50	99,425.9	63,021.3	67.67
	Arc Count 4		
45–30	147,146.8	92,325.7	99.14
50–35	134,939.1	86,619.6	93.01
55–40	123,981.1	80,251.9	86.18
60–45	117,922.5	77,233.1	82.93
65–50	109,651.8	73,832.9	79.28
	Arc Count 7		
45–30	164,085.2	106,586.4	114.45
50–35	152,956.3	99,365.7	106.70
55–40	143,382.8	92,210.3	99.02
60–45	138,346.2	88,923.6	95.49
65–50	130,932.4	84,680.5	90.93

**Table 4:** Effect of the wall thickness (t_min_–t_max_) on the surface area and volume of the TPMS-based structure.

Thickness Min–Max (mm)	Cell 45–30, Arc C. 4		Cell 65–50, Arc C. 4		Cell 45–30, Arc C. 7		Cell 65–50, Arc C. 7	
	Volume (mm^3^)	Volume Fraction (%)	Volume (mm^3^)	Volume Fraction (%)	Volume (mm^3^)	Volume Fraction (%)	Volume (mm^3^)	Volume Fraction (%)
1.4–1.8	78,661.9	84.47	64,162.3	68.90	90,059.9	96.71	72,967.3	78.35
1.6–2.0	85,358.0	91.66	68,967.2	74.06	98,595.2	105.87	78,843.9	84.66
1.8–2.2	92,325.7	99.14	73,832.9	79.28	106,586.4	114.45	84,680.5	90.93
2.0–2.4	98,573.8	105.85	78,433.0	84.22	114,385.8	122.83	90,668.3	97.36
2.2–2.6	105,246.0	113.01	83,781.1	89.97	122,939.0	132.01	96,705.7	103.84

**Table 5:** Comparison of the volumes of the domains of the reference sample and the TPMS geometry.

	MNTZV-159	TPMS
External shell—stainless steel (mm^3^)	87,368	87,368
Heat exchanger—aluminium (mm^3^)	93,126	92,210
Metal hydride (mm^3^)	613,544	614,460

**Table 6:** Counts of the mesh elements in individual domains (in millions of elements).

	MNTZV-159	TPMS
External shell—stainless steel	0.07	0.07
Heat exchanger—aluminium	0.40	3.88
Metal hydride	5.38	10.25
TOTAL	5.82	14.10

**Table 7:** Sensitivity analysis of the mesh for the original MNTZV-159 geometry.

No. of El. (Mil.)	*t*_MH-ave_ (°C)	Δ*t*_MH-ave_ (%)	*t*_MH-max_ (°C)	Δ*t*_MH-max_ (%)	*P*_HE_ (W)
2.28	108.60	0.94	132.10	1.11	333.74
5.82	107.71	0.12	130.90	0.19	333.74
7.72	107.59	0.00	130.66	0.00	333.74

*t*_MH-ave_—average temperature of metal hydride (°C); Δ*t*_MH-ave_—change in the average temperature of metal hydride after changing the mesh size (%); *t*_MH-max_—maximum temperature of metal hydride (°C); Δ*t*_MH-ave_—change in the maximum temperature of metal hydride after changing the mesh size (%); *P*_HE_—heat removed by the heat exchanger (W).

**Table 8:** Sensitivity analysis of the mesh for the TPMS geometry.

No. of El. (Mil.)	*t*_MH-ave_ (°C)	Δ*t*_MH-ave_ (%)	*t*_MH-max_ (°C)	Δ*t*_MH-max_ (%)	*P*_HE_ (W)
6.11	87.14	1.10	115.46	1.27	333.74
10.28	86.36	0.20	114.32	0.27	333.74
14.15	86.19	0.00	114.01	0.00	333.74

**Table 9:** Material properties defined in the numerical calculation.

Material	Parameter	Value
Metal hydride	Bulk Density	3600 kg·m^−3^
	Effective Thermal Conductivity	1 W·m^−1^·K^−1^
	Specific Heat Capacity	500 J·kg^−1^·K^−1^
	Reaction Heat	1.24 MJ·m^−3^
Aluminium	Density	2700 kg·m^−3^
	Thermal Conductivity	237 W·m^−1^·K^−1^
	Specific Heat Capacity	900 J·kg^−1^·K^−1^
Stainless steel	Density	7854 kg·m^−3^
	Thermal Conductivity	20 W·m^−1^·K^−1^
	Specific Heat Capacity	434 J·kg^−1^·K^−1^

**Table 10:** Calculated temperatures in the compared domains.

	MNTZV-159	TPMS	Δ (%)
Average metal hydride temperature (°C)	107.7	86.2	−19.96
Maximum metal hydride temperature (°C)	130.9	114.0	−12.91
Ambient temperature (°C)	20.0	20.0	
Temperature difference *t*_average—_*t*_amb_ (°C)	87.7	66.2	−24.52
Temperature difference *t*_max—_*t*_amb_ (°C)	110.9	94.0	−15.24
Heat power generated in MH (W)	333.7	333.7	

## Data Availability

The original contributions presented in this study are included in the article. Further inquiries can be directed to the corresponding author.
